# Myricetin-Mediated Lifespan Extension in *Caenorhabditis elegans* Is Modulated by DAF-16

**DOI:** 10.3390/ijms140611895

**Published:** 2013-06-04

**Authors:** Christian Büchter, Daniela Ackermann, Susannah Havermann, Sebastian Honnen, Yvonni Chovolou, Gerhard Fritz, Andreas Kampkötter, Wim Wätjen

**Affiliations:** 1 Institute of Agricultural and Nutritional Sciences, Faculty III, Martin-Luther-Universität Halle-Wittenberg, Weinbergweg 22 (Biozentrum), 06120 Halle/Saale, Germany; E-Mails: christian.buechter@googlemail.com (C.B.); susannah.havermann@landw.uni-halle.de (S.H.); 2 Institute of Toxicology, Heinrich-Heine-Universität Düsseldorf, P.O. Box 101007, 40001 Düsseldorf, Germany; E-Mails: daniela.ackermann@uni-duesseldorf.de (D.A.); sebastian.honnen@uni-duesseldorf.de (S.H.); chovolou@uni-duesseldorf.de (Y.C.); fritz@uni-duesseldorf.de (G.F.); andreas.kampkoetter@bayer.com (A.K.); 3 Global Drug Development, Safety and Pharmacokinetics, Bayer Animal Health GmbH, Bayer HealthCare, Building 6700 Monheim, 51368 Leverkusen, Germany

**Keywords:** *C. elegans*, *daf-16*, flavonoid, lifespan, myricetin, insulin-like signalling, oxidative stress, SKN-1

## Abstract

Myricetin is a naturally occurring flavonol found in many plant based food sources. It increases the lifespan of *Caenorhabditis elegans*, but the molecular mechanisms are not yet fully understood. We have investigated the impact of this flavonoid on the transcription factors DAF-16 (*C. elegans* FoxO homologue) and SKN-1 (Nrf2 homologue), which have crucial functions in the regulation of ageing. Myricetin is rapidly assimilated by the nematode, causes a nuclear translocation of DAF-16 but not of SKN-1, and finally prolongs the mean adult lifespan of *C. elegans* by 32.9%. The lifespan prolongation was associated with a decrease in the accumulation of reactive oxygen species (ROS) detected by DCF. Myricetin also decreases the formation of lipofuscin, a pigment consisting of highly oxidized and cross-linked proteins that is considered as a biomarker of ageing in diverse species. The lifespan extension was completely abolished in a *daf-16* loss-of-function mutant strain (CF1038). Consistently with this result, myricetin was also not able to diminish stress-induced ROS accumulation in the mutant. These results strongly indicate that the pro-longevity effect of myricetin is dependent on DAF-16 and not on direct anti-oxidative effects of the flavonoid.

## 1. Introduction

Flavonoids are phenolic plant metabolites that occur ubiquitously in fruit, vegetables, grains, nuts, tea and wine [1–3]. Up to now, over 6000 different compounds have been identified. Myricetin (3,5,7,3′,4′,5′-hexahydroxyflavone) is a major flavonol that is widely distributed in berries, fruit, vegetables, and medicinal herbs.

**Figure 1:**
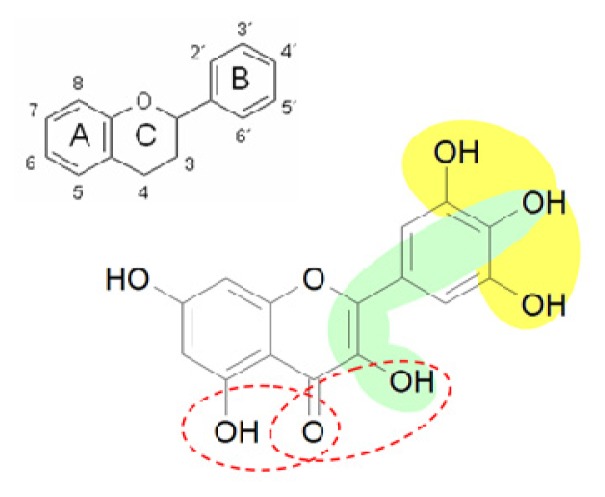
Structure of myricetin. The structural elements of myricetin mediating a direct antioxidative effect are: (i) the catechol groups in ring B (3′OH/4′OH and 4′OH/5′OH) forming a semiquinone radical or ortho quinines after oxidation; (ii) the 4′OH group of ring B in combination with the 2,3 double bond and the 3-OH group forming a quinine methide after oxidation; (iii) the ketogroup (position 4) in combination with 3-OH or 5-OH group chelating redox-active metal ions.

Myricetin (Figure 1) possesses strong anti-oxidative properties due to three OH-groups in ring B (3′,4′,5′-position): Therefore, the compound can scavenge reactive oxygen species (ROS) by oxidation of these hydroxyl groups. Further, direct anti-oxidative effects may also be caused by chelation of redox-active metal ions, e.g., Fe^2+^/Fe^3+^, thereby inhibiting the formation of fenton reaction products like hydroxyl radicals [4].

Flavonoids show both anti-oxidative as well as pro-oxidative properties depending e.g., on intracellular concentration. Pro-oxidative properties of myricetin are caused mutually by the anti-oxidative action of the substance, e.g., reduction of molecular oxygen to superoxide anions (O_2_^−^) or Fe^3+^ to Fe^2+^. Using a deoxyribose degradation assay system, Chobot & Hadacek [4] were able to show that in the presence of ascorbic acid, myricetin exhibited anti-oxidant properties (especially in a complex with iron), while in an ascorbic acid-free system, the pro-oxidant activity prevailed (enhanced if iron was complexed with EDTA). Since pro-oxidant effects of myricetin are known, it is not surprising, that the flavonoid also activates redox-active signalling pathways within the cell: Using a microarray-based pathway analysis, Qin *et al.* [5] showed that activation of the antioxidant response element (ARE) is involved in myricetin-induced modulation of gene expression in human HepG2 hepatoma cells. They propose that myricetin activates this pathway by inhibiting Nuclear factor erythroid 2-related factor 2 (Nrf2) ubiquitination and protein turnover, stimulating Nrf2 expression and kelch-like erythroid cell-derived protein with CNC homology (ECH)-associated protein 1 (Keap1) modification. Myricetin induces pancreatic cancer cell death via inhibition of the phosphatidylinositol 3-kinase (PI3K) signalling pathway [6].

Myricetin is being discussed as an important chemopreventive compound: Physiological concentrations of myricetin significantly inhibited DNA strand breakage induced by both peroxynitrite and its generator 3-morpholinosydnonimine [7]. Myricetin acts as a promising agent for the chemoprevention of skin cancer: Kang *et al.* [8] described that myricetin attenuated the ultraviolet B (UVB)-induced COX-2 expression and skin tumor formation in a mouse skin model by regulating the receptor associated tyrosine kinase Fyn. Furthermore, myricetin was found to inhibit UVB-induced angiogenesis by targeting PI3K in an SKH-1 hairless mouse skin tumorigenesis model. However, it has to be taken into account that the effects of myricetin depend on the bioavailability of the compound: Duthie and Morrice [9] demonstrated that there is an important distinction between the good *in vitro* anti-oxidant effectivity of myricetin and the ability to suppress the oxidation of lipids in hepatic microsomes *in vivo*. In contrast to good anti-oxidative effects *in vitro*, the compound did not significantly affect lipid peroxidation and tissue damage *in vivo*.

Myricetin also influences the glucose metabolism of mammals: Treatment of insulin-resistant rats with this flavonoid affected the phosphorylation of the insulin receptor, insulin receptor substrate-1, Akt and Akt substrate, with subsequent effects on glucose-transporter subtype 4 translocation [10].

Several polyphenols have been reported to increase the lifespan of *Caenorhabditis elegans*, including quercetin, fisetin, resveratrol, catechin, epigallocatechin-gallate or a polyphenolic fraction rich in proanthocyanidines [11–19]. It was also demonstrated that myricetin increases the lifespan of *C. elegans* [20]. Grünz *et al.* observed that myricetin, but no other flavonoid analysed, elongated the lifespan of *mev-1* (*kn1*) mutant animals, suggesting that an anti-oxidant function of a compound is not sufficient for longevity. As a molecular mechanism for the lifespan extension, they suggested a modulation of the insulin/IGF-like signalling pathway. They investigated the activation of the transcription factor DAF-16 (*C. elegans* orthologue of mammalian FoxO transcription factors): Myricetin activates this pathway as detected by an enhanced nuclear localization of DAF-16, thereby increasing the promoter activity of superoxide dismutase 3 (*sod-3*). However, in spite of an increased promoter activity of the anti-oxidative enzyme *sod-3*, no correlation of *daf-16* and the myricetin-induced mitochondrial ROS accumulation and life prolongation was detected [20]. From their experiments, they concluded that myricetin-induced activation of the FoxO orthologue DAF-16 is not the cause of lifespan extension.

### Aim of the study

Since there are several questions remaining concerning the anti-oxidative and lifespan-prolonging effects of myricetin in *C. elegans*, we investigated the kinetics of myricetin uptake in *C. elegans*, the modulation of heat induced intracellular ROS in general (fluorescent probe: DCF) and the effect of myricetin on lipofuscin accumulation, an autofluorescent pigment that is used as a biomarker of ageing. Furthermore, we analysed its effects on DAF-16 and SKN-1 and correlated the modulation of DAF-16 with anti-oxidative effects and ageing. To exclude the possibility that the effects of myricetin were mediated by caloric restriction, we finally analysed the effect of the flavonoid on food uptake by investigating pharyngeal pumping activity and body size.

## 2. Results and Discussion

The flavonoid myricetin increases lifespan of *C. elegans*, but the molecular mechanisms are not fully understood. It has been suggested that the anti-oxidant action of this compound alone is not sufficient to mediate longevity, but that an interaction with distinct intracellular pathways is required. An activation of DAF-16 was described, however no influence of *daf-16* on the life prolonging action induced by myricetin was reported [20]. In contrast to this report, we observed that for the enhancement of lifespan by myricetin the presence of DAF-16 is required.

### 2.1. Uptake of Myricetin by *C. elegans*

**Figure 2:**
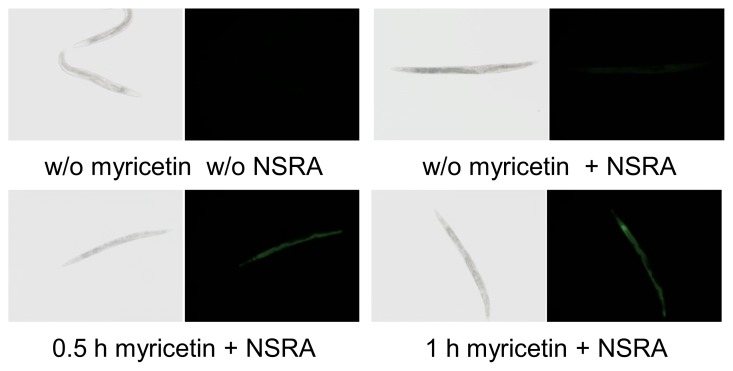

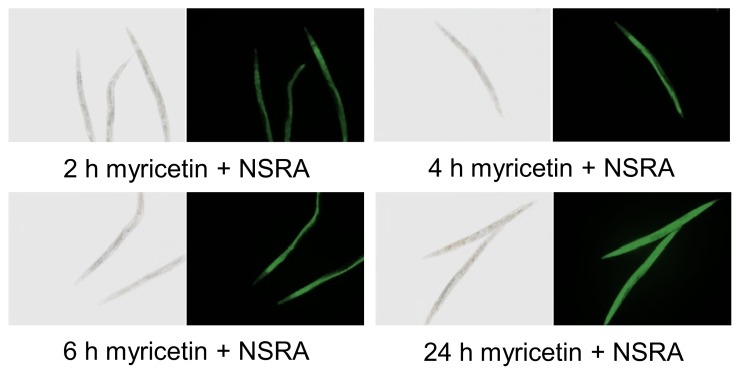
Myricetin is rapidly taken up by *C. elegans*. As early as 30 min after incubation with myricetin (100 μM) followed by two hours of post-treatment with the fluorescence enhancer NSRA (0.2%), a bright fluorescence was detectable in the pharynx and intestine. Representative images of fluorescence (**right**) and brightfield (**left**) micrographs are shown.

Visualization of myricetin uptake in living *C. elegans* by use of the fluorescence enhancer 2-aminoethyl diphenylborinate (Naturstoffreagent A, NSRA) has already been reported and a concentration dependent increase in fluorescence was shown [20]. Here we were able to demonstrate a time dependent increase in myricetin fluorescence in *C. elegans*. Pharyngeal and intestinal fluorescence were clearly visible already after 30 min of myricetin treatment (Figure 2). An increase in fluorescence over time could be observed, with the brightest fluorescence 24 h after myricetin treatment (100 μM). These results indicate that myricetin is rapidly taken up by *C. elegans* and accumulates in the intestine of the nematode in a time dependent manner.

### 2.2. Anti-Oxidative Effects of Myricetin

It is well-known that myricetin exerts anti-oxidative effects in various model systems [21]. To evaluate the anti-oxidative potency of myricetin, we first analysed the radical-scavenging capacity of this flavonoid using the cell free TEAC assay and, furthermore investigated anti-oxidative effects in a cellular system (Hct116 human colon carcinoma cells).

#### 2.2.1. Anti-Oxidative Effects of Myricetin in Cell-Free System/in Hct116 Cells

**Figure 3:**
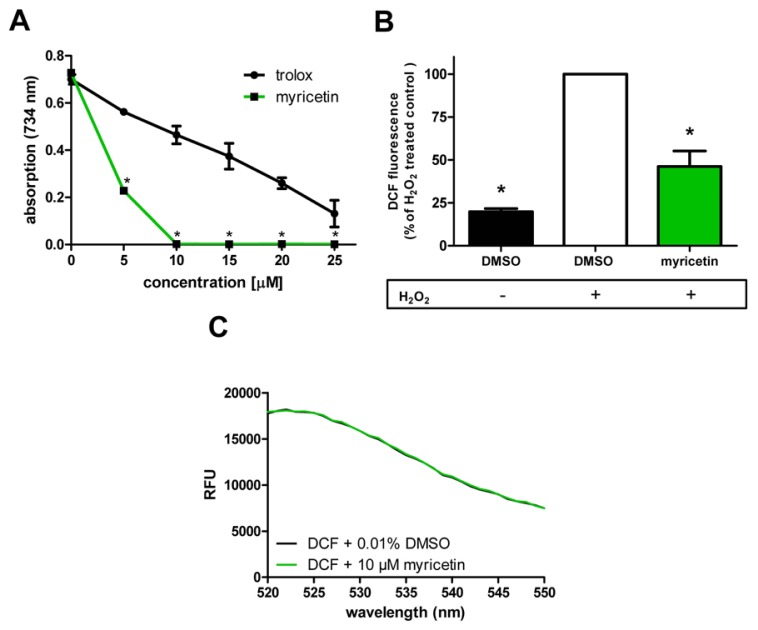
(**A**) Myricetin is a strong radical scavenger in the cell-free TEAC assay; mean values ± SD, *n* = 3, *: *p* < 0.05 *vs.* corresponding trolox value; one way ANOVA; (**B**) Myricetin treatment (50 μM) also reduced intracellular ROS accumulation in Hct116 human colon carcinoma cells after application of 500 μM H_2_O_2_ for 1 h, mean values ± SD (DMSO + H_2_O_2_ set as 100%, the rfu-value of DMSO control before standardization to H_2_O_2_ control was 8447 ± 2121), *n* = 3, *: *p* < 0.05 *vs.* corresponding DMSO + H_2_O_2_ value; one way ANOVA; (**C**) Myricetin (10 μM) did not change the emission spectrum and intensity of DCF fluorescence (measurement without cells to exclude that quenching effects are caused by this flavonoid).

Using the Trolox Equivalent Anti-oxidative Capacity (TEAC) assay, we were able to show that even low concentrations of myricetin possess a radical scavenging capacity (Figure 3A): 5 μM myricetin reduced the absorption of the ABTS radical solution by 68.6%. At a concentration of 10 μM the maximum of radical-scavenging was exceeded. We found that the radical scavenging capacity of myricetin is much stronger than that of the reference compound trolox, a synthetic vitamin E derivative: 5 μM trolox reduced the ABTS radical only by 19.7%; the maximum radical scavenging effect was not reached even at the highest concentration used (25 μM).

Since the anti-oxidative capacity of a compound may differ between cell-free assays and cellular assays, we investigated the anti-oxidative effects of myricetin in Hct116 human colon carcinoma cells: We used DCF, a fluorescent probe for the detection of the overall intracellular ROS content. A pre-incubation with myricetin (50 μM) strongly reduced intracellular ROS accumulation in Hct116 cells after application of oxidative stress (500 μM H_2_O_2_ for 1 h) to 46.1% ± 9.1% of the corresponding control value (DMSO + 500 μM H_2_O_2_) (Figure 3B). An effect of myricetin on basal ROS levels was not detectable in this experimental system (data not shown). The experiment was performed with a concentration of 50 μM myricetin, since in a previous investigation a slight toxicity of this flavonoid was observed at the concentration of 100 μM. The toxicity of myricetin in Hct116 cells (approximate 20% lower ability to reduce MTT compared to control cells) occurred only after 24 h of incubation and not during the short time incubation of the DCF assay, but we decided to work in our experimental systems in a non-toxic range.

To exclude that the effects of myricetin were mediated by physical quenching and not anti-oxidative activity, we analysed the influence of myricetin on the fluorescence intensities of the probe DCF using a monochromator-based fluorescence detector: No interference of myrcetin with DCF was detected (Figure 3C). We could show that myricetin possesses a high direct anti-oxidative capacity both in a cell-free system and in a cellular system.

These results concerning radical-scavenging effects of myricetin in cell-free assay systems are in line with a study by Wang *et al.* [22]. Our results concerning anti-oxidative effects of myricetin in cellular systems are in accordance with studies by Khang *et al.* [23] and Wang *et al.* [22].

#### 2.2.2. Effect of Myricetin on Lipofuscin Accumulation in *C. elegans*

The ageing process is associated with an increased accumulation of highly oxidized and cross-linked proteins called lipofuscin. These modified molecules form insoluble aggregates, which are not degradable by the proteasomal or lysosomal systems. Lipofuscin is a so called “age pigment” and considered as a hallmark of ageing. It is well established that the amount of lipofuscin increases with age, and that the rate of lipfuscin accumulation correlates with the rate of ageing. Moreover, lipofuscin serves as a marker of oxidative stress. Aggregates have been found in various cell types and are associated with the age of these cells [24]. It was shown that these aggregates also accumulate in *C. elegans* with increasing age [25–27]. Lipofuscin is autofluorescent and can be visualized by fluorescence microscopy. We investigated the effect of myricetin on this phenomenon to confirm (i) the anti-oxidative effects of myricetin and (ii) the influence of this flavonoid on the ageing process.

**Figure 4:**
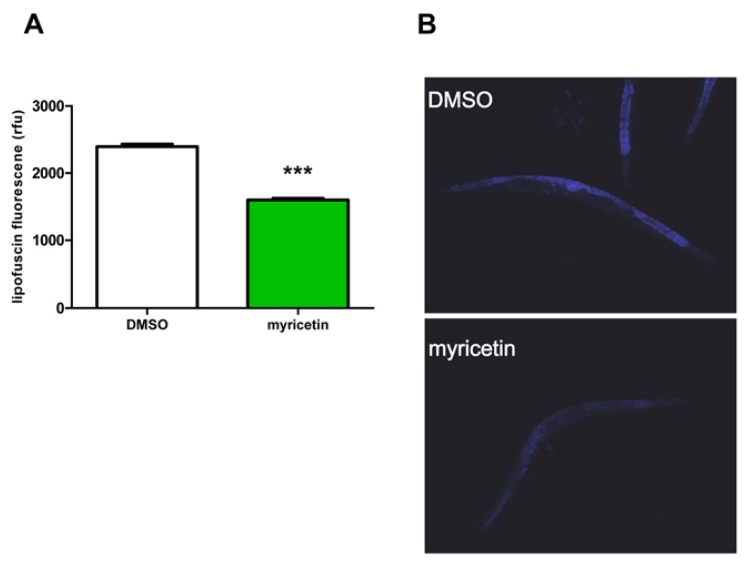
(**A**) Treatment of *C. elegans* (L4 larvae) with myricetin (100 μM) for three days attenuates lipofuscin accumulation; data are mean values ± SEM; *n* ≥ 90 in 4 independent experiments; ***p < 0.001; unpaired Student’s *t*-test. Fluorescence intensities of whole animals (except for head and tail regions) were determined by densitometric analyses; (**B**) Representative images of lipofuscin fluorescence in DMSO-treated nematodes and myricetin-treated nematodes are shown.

Treatment with myricetin reduced the intestinal lipofuscin accumulation in *C. elegans* by 33% ± 1% as compared to the solvent control (DMSO: 2396 ± 36 rfu; myricetin 1602 ± 23 rfu) (Figure 4). A decrease in lipofuscin accumulation was previously shown for various flavonoids, such as quercetin, fisetin and kaempferol [28–30]. In most cases, the reduction of lipofuscin was associated with an increase in lifespan of *C. elegans*. Therefore, the observed reduction of lipofuscin indicates not only a decrease in oxidatively damaged macromolecules; rather it can also point to a decelerated ageing process in myricetin treated nematodes.

#### 2.2.3. Effect of Myricetin on Translocation of DAF-16 and SKN-1

**Figure 5:**
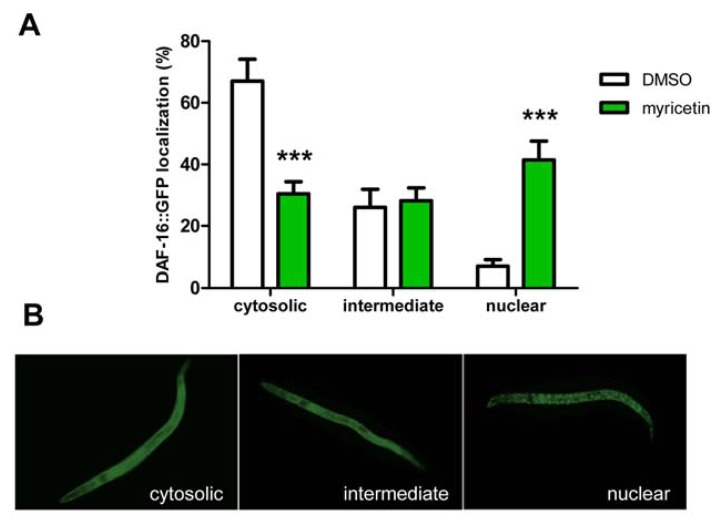

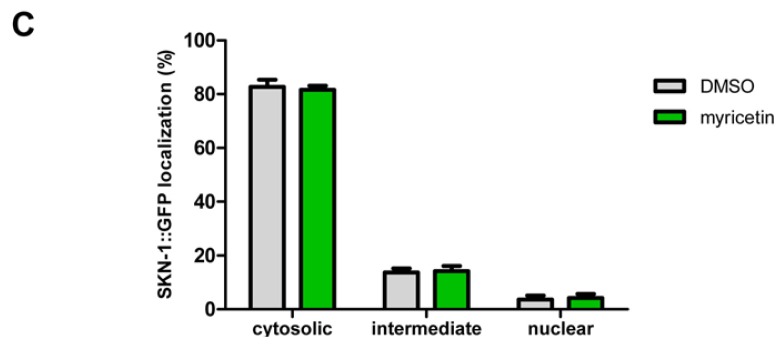
Myricetin induced nuclear translocation of DAF-16::GFP, but not SKN-1::GFP. Nematodes were treated with DMSO or myricetin (100 μM) for 72 h (DAF-16::GFP) or 24 h (SKN-1::GFP), followed by determination of the GFP-localization phenotype. (**A**) Activation of DAF-16 nuclear translocation by myricetin; mean ± SEM, *n* > 200 in four independent experiments; ***p < 0.001 *vs.* corresponding DMSO value; two-way ANOVA; (**B**) Representative images of transgenic strain TJ356 with cytosolic (**left**) intermediate (**center**) and nuclear (**right**) DAF-16::GFP localization; (**C**) Myricetin showed no effect on the SKN-1 signalling pathway; mean ± SEM, *n* > 90 in four independent experiments, *p* > 0.05 *vs.* corresponding DMSO value; two-way ANOVA.

Certain flavonoids protect against oxidative stress either by direct radical scavenging or indirectly by increasing the stress resistance of the organism for example by induction of anti-oxidative enzymes. In mammalian cells, this indirect effect is often mediated via the insulin-like signalling pathway or the redox-active Nrf2/ARE signalling pathway. To analyse the effect of myricetin on these signalling pathways, we investigated if this flavonoid induces a nuclear translocation of the corresponding transcription factors DAF-16 (homologue to the mammalian transcription factor FoxO) and SKN-1 (homologue to the mammalian transcription factor Nrf2) by using transgenic strains expressing the fusion proteins DAF-16::GFP and SKN1::GFP, respectively. When GFP fluorescence was detected mainly in the nuclei (in case of SKN-1 exclusively in intestinal nuclei), the signalling pathway of the nematode was classified as active (see Figure 5C). Regarding the insulin-like signalling pathway, myricetin induced an increase in nuclear DAF-16::GFP translocation (relative DAF-16::GFP localization: DMSO: 6% ± 2%; myricetin: 41% ± 7%) and a reduction in the cytosolic DAF-16::GFP fraction (DMSO: 65% ± 8%; myricetin: 30% ± 4%), respectively (Figure 5A). A nuclear translocation of DAF-16 or target gene expression was reported for different flavonoids, e.g., quercetin [28], apigenin [31], fisetin [29], kaempferol [29] and epigallocatechin-gallate [16]. A shift from cytosolic to nuclear localization of DAF-16 by myricetin (20% cytosolic compared to 70% cytosolic in control nematodes) was previously reported [20].

Furthermore, we investigated the effect of myricetin on the Nrf2/ARE signalling pathway in *C. elegans* (=SKN-1). Nrf2 is a redox-active transcription factor that binds to the antioxidant responsive element (ARE), a distinct DNA motif in the promoter region of various antioxidant and phase II genes. Due to induction of antioxidant enzymes, this signalling pathway is a key cellular defense mechanism against oxidative stress and hence activation of this pathway is associated with beneficial effects. The Nrf2 signalling pathway builds up a prolonged defense system compared to the uptake of compounds that act only by scavenging radicals. An activation of the SKN-1 signalling pathway by flavonoids was reported previously for baicalein [32] and epigallocatechin-gallate [33]. However, treatment of *C. elegans* with myricetin (100 μM) did not change the localization of the transcription factor showing that (i) SKN-1 is not activated by this flavonoid (Figure 5C) and (ii) flavonoids need to possess distinct structural features to activate this signalling pathway.

#### 2.2.4. Requirement of DAF-16 for the Anti-oxidative Effects of Myricetin in *C. elegans*

Flavonoids can either modulate oxidative stress directly by radical scavenging or indirectly by increasing the stress resistance of the organism e.g., by induction of anti-oxidative enzymes via activation of protective signalling pathways such as SKN-1 or DAF-16. We showed that myricetin strongly induced the nuclear translocation of DAF-16, but had no influence on SKN-1. For this reason we investigated the effect of myricetin on stress-induced ROS accumulation in wild-type (N2) as well as in *daf-16* (*mu86*) mutant nematodes. In this experimental setting, an increased formation of ROS in the nematodes was provoked (measurement at 37 °C) as detected by the fluorescent probe DCF.

**Figure 6:**
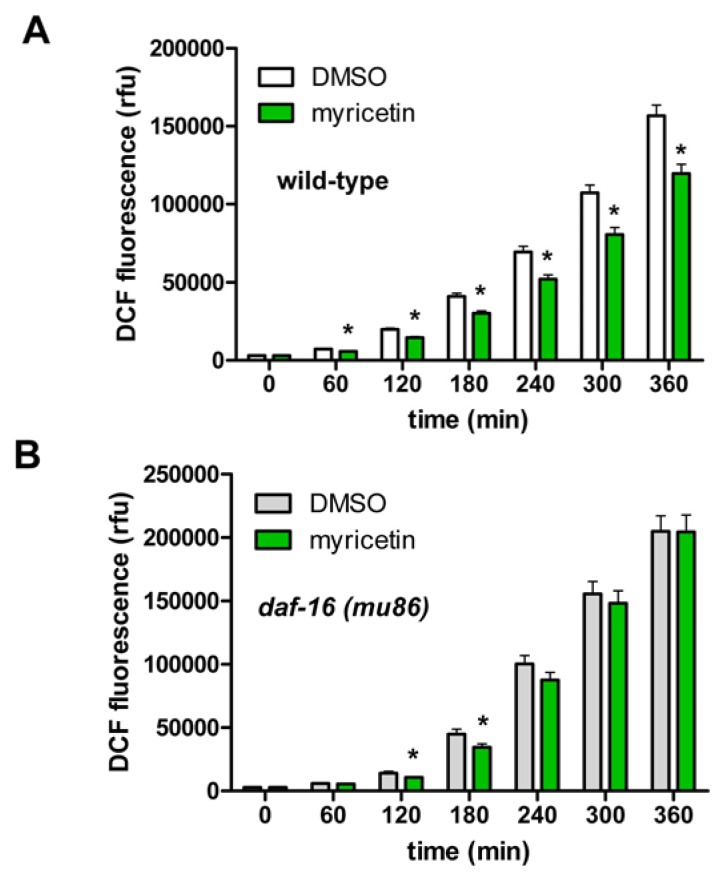
(**A**) Myricetin treatment (100 μM) reduced ROS accumulation in wild-type (N2) nematodes at 37 °C. The fluorescence intensity of DCF (rfu) was used as a marker for intracellular ROS; data are mean values ± SEM, *n* = 64 in four independent experiments; *p < 0.05, unpaired Student’s *t*-test; (**B**) The reduction in ROS accumulation was abolished in *daf-16* (*mu86*) mutant animals with the exception of 120 min and 180 min (**B**); data are mean values ± SEM, *n* = 64 in four independent experiments; * *p* < 0.05 *vs.* corresponding DMSO control, unpaired Student’s *t*-test.

In analogy to the results obtained in the mammalian cell system, incubation with myricetin strongly diminished ROS accumulation in wild-type nematodes (Figure 6A). This result is comparable to data of Grünz *et al.* [20] who investigated the effect of myricetin on the formation of mitochondrial ROS under basal conditions in *C. elegans*. In contrast to this group, we used H_2_DCF-DA in order to show alterations in ROS concentration in general during the application of heat stress.

Since we have shown that myricetin stimulated an enhanced nuclear translocation of DAF-16, we further investigated whether the presence of DAF-16 is necessary for the anti-oxidative effects of myricetin in *C. elegans*. To this end, we analysed stress induced ROS accumulation in *daf-16* (*mu86*) mutant nematodes. Loss of function of the FoxO transcription factor DAF-16 nearly completely abolished the protective effect of myricetin (Figure 6B).

The slight reduction of oxidative stress which was detectable only at the early time points (120 + 180 min) may be due to a DAF-16-independent antioxidative effect of myricetin, e.g., radical scavenging. This result clearly shows that functional DAF-16 is necessary for a prolonged anti-oxidative effect of myricetin; the radical scavenging activity of the flavonoid alone seems not to be sufficient to decrease the formation of ROS in our experimental system efficiently for a prolonged time span.

### 2.3. DAF-16 Is Required for Myricetin-Induced Prolongation of Lifespan

**Figure 7:**
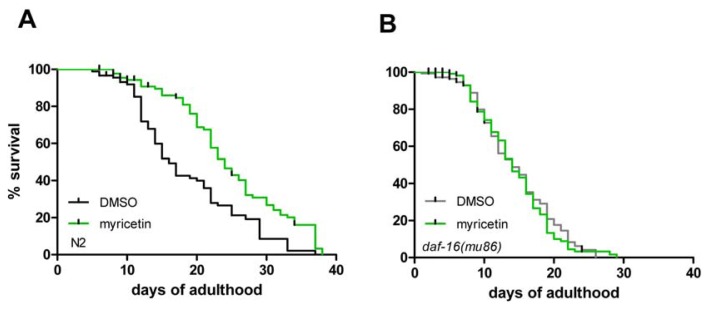
(**A**) Myricetin treatment (100 μM) during the whole adult lifespan extended mean lifespan of wild-type (N2) *C. elegans*; (**B**) Lifespan extension was abolished in a strain bearing the *daf-16* (*mu86*) loss of function mutation. Lifespan data are shown in Table 1; Kaplan-Meier survival analysis.

Next, we investigated if myricetin modulates the lifespan of *C. elegans*: Wild-type nematodes were treated with myricetin (100 μM) during their complete adult lifetime. As shown in Figure 7A and Table 1, myricetin extended the mean adult lifespan by 32.9%: lifespan of DMSO-treated nematodes was 18.7 ± 0.9 days, while myricetin-treated *C. elegans* had a mean lifespan of 24.8 ± 0.9 days. These data are in line with observations showing that myricetin prolongs lifespan in wild-type nematodes by 18% [20]. Several anti-oxidative polyphenols have already been identified to extend the lifespan of *C. elegans*, e.g., curcurmin [34], quercetin [11] and baicalein [32].

However, the myricetin-mediated prolongation of lifespan in wild-type *C. elegans* was completely abolished in *daf-16* (*mu86*) mutant animals (Figure 7B and Table 1). This effect indicates that the transcription factor DAF-16 is responsible for myricetin mediated lifespan extension in wild-type nematodes, probably due to a modulation of antioxidant and stress responsive genes. Moreover, the data also indicate that lifespan extension by myricetin is largely independent of its radical scavenging properties. In contrast to these results, Grünz *et al.* [20] reported that myricetin mediates a lifespan extension independent of *daf-16*. This discrepancy could be explained by different experimental settings, which may alter the outcome of the experiment. In our experiments we used liquid culture medium and a temperature of 25 °C for the treatment, while Grünz *et al.* used agar plates and 20 °C for their experimental setup.

### 2.4. Myricetin Has No Effect on Resistance against Thermal Stress

**Figure 8:**
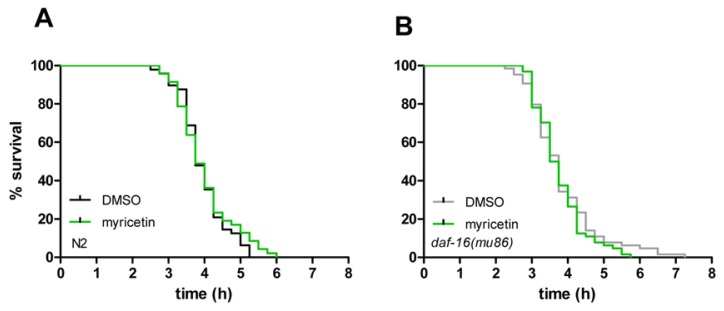
Myricetin treatment (100 μM) had no effect on the resistance to thermal stress of wild-type (**A**) and *daf-16* (*mu86*) nematodes (**B**). The corresponding survival data are summarized in table 2; Kaplan-Meier survival analysis.

Since myricetin reduces oxidative stress and increases lifespan in *C. elegans*, we investigated if this flavonoid also mediates resistance to thermal stress. Thermal stress (37 °C) is known to be lethal in *C. elegans*. We analysed the effect of myricetin on the tolerance of *C. elegans* against thermal stress using the semi-automated SYTOX Green assay: 50% of the nematodes were counted dead after 3.75 h; after 5.25 h at 37 °C, no animal was alive (Figure 8A). In spite of the protective effects of myricetin against ROS accumulation, the flavonoid failed to elicit any protective effects against the lethal heat stress: Mean survival time of DMSO treated wild-type individuals was 3.94 ± 0.09 h compared to 4.01 ± 0.12 h survival time of myricetin-treated nematodes (Table 2). Using the *daf-16* (*mu86*) mutant strain, similar results were obtained: myricetin again failed to protect against thermal stress (Figure 8B). Mean survival time of DMSO treated *daf-16* (*mu86*) nematodes was 3.84 ± 0.12 h compared to 3.76 ± 0.09 h survival of animals treated with myricetin (Table 2). Taken together, in contrast to lifespan extending effects of myricetin (depending at least in part on DAF-16) this flavonoid failed to protect against heat stress in both wild-type and *daf-16* (*mu86*) nematodes. These results show that an increase in lifespan and protection from ROS are not necessarily associated with a protection from heat stress in spite of the thermal-mediated generation of ROS.

### 2.5. Effects of Myricetin Are Not Mediated by Caloric Restriction

**Figure 9:**
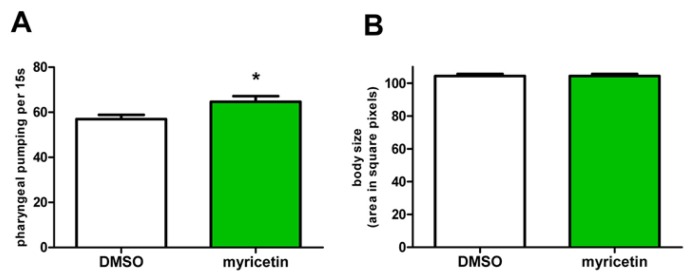
Effects of myricetin are not mediated by caloric restriction: Myricetin treatment (100 μM) for 2 days (**A**) and 4 days (**B**) did not reduce food uptake (**A**) and body size (**B**) of the nematodes; (**A**) data are mean values ± SEM, *n* = 15 in 3 independent experiments; *p < 0.05; unpaired Student’s *t*-test; (**B**) data are mean values ± SEM, *n* ≥ 101 in 3 independent experiments; *p >* 0.05; unpaired Student’s *t*-test.

It is well-known, that caloric restriction results in a prolongation of lifespan [35,36]. To exclude that the effects of myricetin on lifespan of *C. elegans* were mediated by a restriction in food uptake due to e.g., bitter taste of the compound, we determined food uptake of the nematodes by analysing the pharyngeal pumping activity. Since myricetin-treated nematodes showed no reduction in pharyngeal pumping activity compared to control nematodes (Figure 9A), we conclude that the myricetin induced effects were not due to caloric restriction. Further, this hypothesis is supported by an analysis of the body size of the nematodes, which is another indicator of caloric restriction. Myricetin had also no effect on the body size of *C. elegans* (Figure 9B). Therefore, we conclude that myricetin exerts its prolonging effect on lifespan independent of caloric restriction.

## 3. Experimental Section

### 3.1. *C. elegans* Strains and Maintenance

The strains used in this study were N2 var. Bristol, CF1038 *[daf-16(mu86) I.]*, TJ356 *[zIs356 IV (pdaf-16-daf-16::gfp; rol-6)]* and LD001 *[Is007 (skn-1::gfp; rol-6)]*. Some strains were provided by the *Caenorhabditis* Genetics Center (CGC), which is funded by NIH Office of Research Infrastructure Programs (P40 OD010440). Strains were maintained on nematode growth medium (NGM) agar plates at 20 °C containing a lawn of Escherichia coli var. OP50 (provided by the CGC) as the food source, as described elsewhere [37]. Treatment of *C. elegans* with the test compound was performed in 2 mL of liquid NGM containing 1% (*w*/*v*) bovine serum albumin (Sigma, Deisenhofen, Germany), 50 μg/mL streptomycin (Sigma) and 1 × 10^9^ OP50-1/mL (provided by the CGC) in 35 mm petri dishes (Greiner Bio-One, Frickenhausen, Germany). Myricetin (Extrasynthese, Genay, France) stock solution was prepared with the solvent DMSO (Merck) in a concentration of 100 mM. In all assays, myricetin was used in a final concentration of 100 μM and 0.1% (*v*/*v*) DMSO was used as the solvent control. Age synchronous animals were obtained by bleaching of gravid adults. Briefly, gravid adults were rinsed off NGM agar plates with liquid NGM, collected in 0.5 mL liquid NGM and mixed with 0.5 mL bleaching solution (50% 5 M NaOH/50% NaClO). Worms were then incubated at room temperature for three minutes, occasionally vortexed, pelleted by centrifugation (5000 rpm/4 °C/1 min) and the supernatant was discarded. The worm pellet was washed three times with liquid NGM and transferred to fresh NGM agar plates (containing OP50 lawn) and maintained for three days at 20 °C to obtain an age synchronous population of mainly L4 larvae.

### 3.2. *In Vivo* Visualization of Myricetin: Fluorescent Staining with 2-Aminoethyl Diphenylborinate

*In vivo* visualization of myricetin in *C. elegans* was performed with slight modifications as described elsewhere [20]. Briefly, randomly picked 4 d old young adult animals were placed in liquid NGM ± 100 μM myricetin and 2% heat killed *E. coli* OP50 for the indicated time (0 min, 5 min, 30 min, 1 h, 2 h, 4 h, 6 h and 24 h) at 20 °C. Then, worms were transferred into liquid NGM containing 2% heat killed *E. coli* OP50 and 0.2% (*w*/*v*) 2-aminoethyl diphenylborinate (Naturstoffreagent A, NSRA) (Sigma, Deisenhofen, Germany) for 2 h. The enhanced fluorescence of myricetin in *C. elegans* was detected by fluorescence microscopy (excitation wavelength 460–495 nm; emission wavelength 510–550 nm; Olympus BX43). Experiments were repeated two times.

### 3.3. Determination of Lipofuscin Accumulation

Over the lifetime of *C. elegans*, the autofluorescent pigment lipofuscin accumulates in gut granules and serves as an established marker of ageing [26]. Randomly picked L4 larvae were placed in liquid NGM ± 100 μM myricetin as described above and incubated for 72 h at 20 °C, followed by 24 h of incubation in compound free medium. During the incubation period, worms were transferred to fresh culture media daily. The lipofuscin fluorescence of seven days old worms was detected by fluorescence microscopy (excitation wavelength 360–370 nm; emission wavelength 420–460 nm; Olympus BX43, Olympus, Hamburg, Germany) and analysed densitometrically (ImageJ, National Institutes of Health, Bethesda, MD, USA). Therefore, the fluorescence of the whole body of each animal (except for head and tail regions) was determined and the background fluorescence was subtracted. The experiment was repeated four times.

### 3.4. Intracellular Localization of DAF-16::GFP and SKN-1::GFP

Transgenic strain TJ356 *[zIs356 IV (pdaf-16-daf-16::gfp; rol-6)]* was used to detect the intracellular localization of GFP tagged DAF-16 protein. Therefore, embryos of this strain were placed in liquid NGM ± 100 μM myricetin directly after the synchronization procedure and incubated for 72 h at 20 °C. Subsequently, three days old larvae were placed on microscope slides, covered with cover slips and the cellular localization of DAF-16::GFP was detected by fluorescence microscopy (excitation wavelength 460–495 nm; emission wavelength 510–550 nm; Olympus BX43). The experiment was repeated four times. Strain LD001 *[Is007 (skn-1::gfp; rol-6)]* was used to observe intestinal nuclear localization of SKN-1::GFP. Three days old larvae and young adult animals were placed in liquid NGM ± 100 μM myricetin as described above for 24 h at 20 °C. Thereafter, animals were transferred on microscope slides, covered with cover slips and the cellular localization of SKN-1::GFP was detected by fluorescence microscopy (excitation wavelength 460–495 nm; emission wavelength 510–550 nm; Olympus BX43). Experiments were repeated four times.

### 3.5. Lifespan Assays

Lifespan analyses were performed with N2 and CF1038 *[daf-16(mu86) I]*. About 30–50 L4 larvae per group and experiment were placed in liquid NGM ± 100 μM myricetin as described above and incubated at 25 °C. The starting day in liquid culture was considered as day 0 of the lifespan. Nematodes were transferred daily to new culture dishes during their fertile period to prevent overcrowding and to discriminate the test nematodes from their progeny. After the fertile period, worms were transferred to fresh medium every other day. Worms were scored as dead when they did not respond to gentle prodding with a flexible glass rod and when they showed no pharyngeal pumping movement. Lost worms and animals containing hatched larvae were censored. Experiments were repeated three times and Kaplan-Meier survival analysis was used to detect statistical differences (IBM SPSS 19).

### 3.6. Measurement of Intracellular ROS Accumulation in *C. elegans*

The fluorescent probe H_2_DCF-DA (2′,7′-dichlorodihydrofluorescein–diacetate; Sigma) was used to detect the intracellular ROS level in living individual nematodes. H_2_DCF-DA is able to freely cross cell membranes, however, after entering the cell, non-fluorescent H_2_DCF-DA becomes deacetylated to form the non-fluorescent derivative H_2_DCF that is trapped within the cell. Then H_2_DCF can quickly be oxidized by intracellular ROS to form fluorescent DCF that can be measured in a fluorescence spectrophotometer (excitation wavelength 485 nm; emission wavelength 535 nm). The fluorescence intensity correlates with the intracellular amount of ROS. The experiment was performed as described elsewhere [28]. Briefly, L4 larvae were incubated in liquid NGM ± 100 μM myricetin or 0.1% DMSO for 48 h at 20 °C. During the incubation period, worms were transferred to fresh culture media daily. After 48 h, all animals were transferred into PBST (PBS with 0.1% Tween 20) for one hour. Then single worms were transferred individually in 1 μL PBST into each well of a 384-well plate (384-well μClear® plate, Greiner Bio-One, Frickenhausen, Germany) containing 7 μL PBS. For measurement of the background fluorescence (without nematodes), 8 μL PBS were added into one column of the 384-well plate (16 wells). Subsequently, when all animals were transferred, 2 μL H_2_DCF-DA (250 μM in PBS) were added into each well to obtain a final concentration of 50 μM H_2_DCF-DA. A black backing tape (Perkin Elmer) was applied to the top of the plate to avoid evaporation. ROS accumulation was induced by thermal stress at 37 °C and recorded every 15 min for a period of 12 h in a fluorescence spectrophotometer (Wallac Victor^2^ 1420 Multilabel-Counter, Perkin Elmer, Wellesley, MA, USA). The experiment was repeated four times.

### 3.7. Cell Culture and Measurement of ROS Accumulation in Hct116 Cells

Human colon carcinoma cell line Hct116 was maintained in DMEM high glucose (Gibco, Invitrogen, Carlsbad, CA, USA) supplemented with 10% heat inactivated FCS and 100 U/mL penicillin/100 μg/mL streptomycin (Gibco) at 37 °C and 5% CO_2_ in a humidified incubator (Binder, Tuttlingen, Germany).

The fluorescent probe H_2_DCF-DA (2′,7′-dichlorodihydrofluorescein–diacetate; Sigma), was used to detect intracellular accumulation of ROS. Briefly, Hct116 cells were seeded into 6-well plates with a density of 5 × 10^5^ cells/well and allowed to attach for 24 h. Subsequently, Hct116 cells were treated with 50 μM myricetin or DMSO as solvent control in serum-free DMEM for 4 h. Then, cells were washed with medium, incubated with 10 μM H_2_DCF-DA for 15 min, washed again and treated with 500 μM H_2_O_2_ to induce the generation of ROS. Afterwards, cells were washed with phosphate buffered saline (PBS) and the DCF fluorescence was determined by flow cytometry (Accuri C6, Accuri Cytometers, St. Ives, Cambs, UK).

### 3.8. Measurement of Fluorescence Emission Spectra

Emission spectra of oxidized DCF (dichlorofluorescein; Sigma) in the presence of myricetin (10 μM) or the equal volumen DMSO (0.01%) as solvent control were determined in a monochromator-based microplate reader (Synergy Mx; BioTek, Bad Friedrichshall, Germany) to exclude quenching effects of the flavonoid in the DCF assay. A solution of oxidized DCF in PBS was prepared, then myricetin or DMSO were added to yield an end-concentration of 10 μM myricetin and 0.01% DMSO, respectively. Samples (100 μL) were transferred into black 96 well plates (Nalge Nunc International, Thermo Fischer Scientific Inc., Waltham, MA, USA) and the fluorescence emission spectra were recorded from 520 to 550 nm (1 nm intervals) with an excitation wavelength of 475 nm (*n* = 2).

### 3.9. Trolox Equivalent Anti-Oxidative Capacity (TEAC) Assay

The TEAC assay is a cell-free method for the measurement of radical scavenging properties of compounds [38]. The principle of this reaction is the reductive conversion of a stable, blue-green radical by an antioxidant. The solution becomes decolorized when an antioxidant is added and can be quantified photometrically. Substances without antioxidant activities show no decolorization of the radical solution. Thus, the decolorization of the radical solution indicates the anti-oxidative capacity of a compound which is compared to the potency of the reference substance Trolox (Calbiochem, Merck, Darmstadt, Germany), which is a synthetic vitamin E derivative. The radical solution was prepared the day before use and consists of a 1:1 mixture of 4.9 mM APS and 14 mM ABTS (Sigma, Deisenhofen, Germany) and is stored in the dark at room temperature. The absorption of this solution (1.4 at a wavelength of 734 nm) was adjusted by dilution with 80% (*v*/*v*) ethanol. The reference- and test-substances were measured in a concentration range from 0 to 25 μM by mixing 500 μL of the radical solution with 500 μL of the test solution. The radical scavenging activity was measured after two minutes at 734 nm with a spectrophotometer (Lambda 25 UV/VIS Spectrometer, Perkin Elmer, Wellesley, MA, USA). Three independent trials were performed.

### 3.10. Thermotolerance Assay

Survival of individual nematodes at 37 °C was monitored with an assay described previously [39,40] with slight modifications. After treating L4 larvae for 48 h with 100 μM myricetin or 0.1% DMSO (daily transfer of the animals into fresh culture medium), worms were washed in PBST for 1 h and then individually transferred in 1 μL PBST into the wells of a 384-well plate (384-well μClear® plate, Greiner Bio-One, Frickenhausen, Germany) containing 9 μL PBS. Following the complete transfer of the nematodes, 10 μL of 2 μM SYTOX^®^ Green Nucleic Acid Stain (Molecular Probes Inc., Leiden, The Netherlands) in PBS were added to each well and the plate was sealed using black backing tape (Perkin Elmer, Wellesley, MA, USA) to avoid evaporation. SYTOX^®^ Green Nucleic Acid Stain is unable to pass the membranes of intact cells. However, thermal stress causes an impairment of the cellular membrane, thereby enabling the dye to enter the cells. There the dye binds to DNA and exerts a bright fluorescence that can be used as a marker for cellular damage and thus for the viability of individual nematodes [39]. The fluorescence intensity was determined with a fluorescence spectrophotometer (Wallac Victor^2^ 1420 Multilabel-Counter, Perkin Elmer Wellesley, MA, USA) and was recorded every 15 min for 12 h (excitation wavelength 485 nm; emission wavelength 535 nm). The fluorescence curve of each nematode was calculated and the individual cut off value was determined by multiplying the average fluorescence of the first four measurements by the factor 3. The time point when the fluorescence exceeded the cut off value for each well was defined as the point of death of the respective nematode. The factor 3 in the calculation of the cut off value was previously shown to be adequate [39]. Survival curves and mean survival times were determined from these individual times of death (Kaplan-Meier survival analysis, IBM SPSS 19). Experiments were repeated at least three times.

### 3.11. Pharyngeal Pumping Assay

For the uptake of food from the surrounding environment, *C. elegans* permanently shows pharyngeal pumping movements to filter bacteria and discard the remaining fluid. Therefore, the frequency of pharyngeal movements serves as a indicator of the feeding status of *C. elegans*; e.g., *eat-2* mutants suffer from caloric restriction due to a reduced pharyngeal pumping activity [41]. For testing an influence of myricetin on the feeding behavior of the nematodes, wild-type L4 larvae were treated with 100 μM myricetin or 0.1% DMSO in liquid medium for 48 h at 20 °C. During the incubation period, worms were transferred to fresh culture media daily. Subsequent to the treatment, pharyngeal movement of worms was counted for 15 s and repeated three times with the corresponding worm, using a stereo microscope (Stemi 2000-C, Zeiss, Göttingen, Germany). Each experiment was performed with 5 nematodes per group and the experiment was repeated three times.

### 3.12. Determination of Body Size

About 30 wild-type L4 larvae per experiment and group were randomly selected, transferred into liquid NGM ± 100 μM myricetin and incubated for 96 h at 20 °C. During the incubation period, worms were transferred to fresh culture media daily. Images of individual nematodes were prepared (Olympus BX43, Olympus, Hamburg, Germany) and the body size was determined by measuring the area of each worm (ImageJ, National Institutes of Health, Bethesda, MD, USA). The experiment was repeated three times.

### 3.13. Statistical Analysis

Statistical analysis was performed with SPSS 19 (IBM) and Prism 5 (GraphPad) software and the results are presented as mean ± SD (*in vitro* experiments) and mean ± SEM (*in vivo* experiments). Statistical significance was determined by Student’s *t*-test, one-way ANOVA or two-way ANOVA with Bonferroni post-test. Lifespan analysis was performed using Kaplan-Meier survival analysis; animals that were lost, killed or showed internal hatching were censored. The minimum level of significance was set to *p* < 0.05.

## 4. Conclusions

The lifespan prolongation in *C. elegans* caused by myricetin is associated with anti-oxidative effects, including reduced accumulation of ROS and a decrease in lipofuscin aggregates but not with a reduction in food uptake (caloric restriction). The effect of myricetin on lifespan was completely abolished in a *daf-16* loss-of-function mutant strain. Consistently, the effect of myricetin on stress-induced ROS accumulation was also largely blocked in this mutant strain. These results strongly indicate that the effect of myricetin on lifespan in *C. elegans* is dependent on DAF-16 and not mediated by the direct anti-oxidative effects of this flavonoid.

## Figures and Tables

**Table 1:** Summary of the lifespan data depicted in Figure 7.

Strain	Exp.	Treatment	Mean lifespan (days)	SE	*n* (censored)	% Difference	*p* value
wild-type	3	DMSO	18.7	0.9	90 (20)	32.9	<0.001
wild-type	3	myricetin	24.8	0.9	90 (18)		

*daf-16 (mu86)*	3	DMSO	15.1	0.5	153 (46)	0.8	0.788
*daf-16 (mu86)*	3	myricetin	15.2	0.5	150 (46)		

**Table 2:** Myricetin treatment (100 μM) had no effect on the resistance to thermal stress of wild-type (A) and *daf-16* (*mu86*) nematodes: Summary of the heat stress survival data depicted in Figure 8.

Strain	Exp.	Treatment	Mean lifespan (hours)	SE	*n*	% Difference	*p* value
wild-type	3	DMSO	3.94	0.09	48	1.8	0.473
wild-type	3	myricetin	4.01	0.12	47		

*daf-16 (mu86)*	4	DMSO	3.84	0.12	64	2.1	0.403
*daf-16 (mu86)*	4	myricetin	3.76	0.09	64		
